# Toxin Instability and Its Role in Toxin Translocation from the Endoplasmic Reticulum to the Cytosol

**DOI:** 10.3390/biom3040997

**Published:** 2013-12-10

**Authors:** Ken Teter

**Affiliations:** Burnett School of Biomedical Sciences, College of Medicine, University of Central Florida, 12722 Research Parkway, Orlando, FL 32826, USA; E-Mail: kteter@mail.ucf.edu; Tel.: +1-407-882-2247; Fax: +1-407-384-2062

**Keywords:** AB toxin, cholera toxin, cytolethal distending toxin, endoplasmic reticulum-associated degradation, exotoxin A, pertussis toxin, ricin toxin, Shiga toxin, toxin structure, ubiquitin-independent degradation

## Abstract

AB toxins enter a host cell by receptor-mediated endocytosis. The catalytic A chain then crosses the endosome or endoplasmic reticulum (ER) membrane to reach its cytosolic target. Dissociation of the A chain from the cell-binding B chain occurs before or during translocation to the cytosol, and only the A chain enters the cytosol. In some cases, AB subunit dissociation is facilitated by the unique physiology and function of the ER. The A chains of these ER-translocating toxins are stable within the architecture of the AB holotoxin, but toxin disassembly results in spontaneous or assisted unfolding of the isolated A chain. This unfolding event places the A chain in a translocation-competent conformation that promotes its export to the cytosol through the quality control mechanism of ER-associated degradation. A lack of lysine residues for ubiquitin conjugation protects the exported A chain from degradation by the ubiquitin-proteasome system, and an interaction with host factors allows the cytosolic toxin to regain a folded, active state. The intrinsic instability of the toxin A chain thus influences multiple steps of the intoxication process. This review will focus on the host–toxin interactions involved with A chain unfolding in the ER and A chain refolding in the cytosol.

## 1. AB Protein Toxins

**Figure 1:**
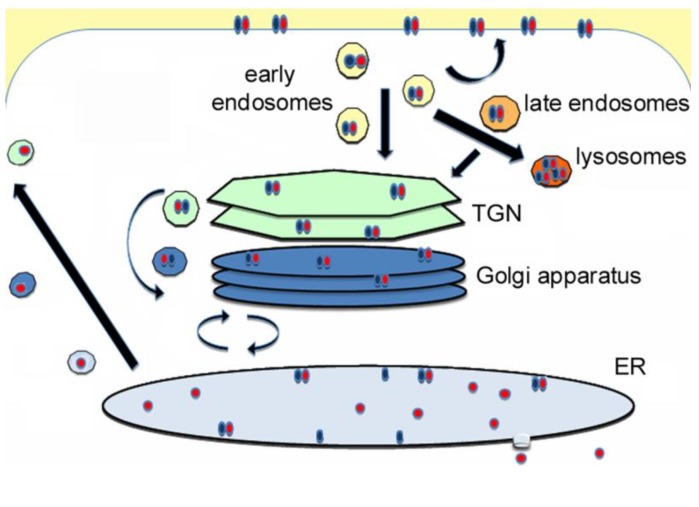
Intracellular toxin trafficking. The general trafficking and translocation itinerary for AB-type, endoplasmic reticulum (ER)-translocating toxins is shown. These toxins bind to distinct surface receptors and are internalized by a variety of endocytic mechanisms. The internalized toxin is recycled to the plasma membrane, directed to the lysosomes for degradation, or delivered to the trans-Golgi network (TGN) en route to the ER translocation site. Vesicle-mediated transport to the TGN can originate from the early or late endosomes, depending on which toxin is present. Likewise, multiple retrograde transport pathways can deliver the toxin from the TGN to the ER. The toxin may cycle between the Golgi and ER until the catalytic subunit dissociates from the rest of the toxin and shifts to an unfolded conformation which triggers its export to the cytosol in a process involving the quality control system of ER-associated degradation. Some of the free, ER-localized A chain escapes ER-associated degradation (ERAD) and is secreted back into the medium via Golgi and TGN intermediates. In most cell types, trafficking from the cell surface to the ER is very inefficient: the majority of internalized toxin is routed to the lysosomes, and only around 10% of surface-bound toxin reaches the ER [9,10,11,12,13,14,15,16]. Thus, ectopic expression of an ER-localized A chain via transfected cultured cells, transformed yeast, or microsomal transcription/translation systems is often used for toxin translocation studies.

AB protein toxins are produced by Gram-negative and Gram-positive bacterial pathogens as well as some plants. All AB toxins have an enzymatically active A moiety and a cell-binding B moiety. These two components can be part of a single polypeptide chain, can be assembled from two different proteins, or can be arranged in a multimeric complex involving several B subunits (*e.g.*, the AB_5_ subfamily of toxins) or even multiple, distinct A chains with several B subunits (*i.e.*, the A_3_B_7_ and A_4_B_8_ arrangements of anthrax toxin). All AB toxins have intracellular targets, yet they are released into the extracellular environment and initially contact the surface of the host cell. The toxins then enter the cell by receptor-mediated endocytosis, but at this stage of intoxication they are still sequestered from their cytosolic targets by a membrane-bound compartment. Some AB toxins have an intrinsic pore-forming capacity that is triggered by the low pH of the acidified endosomes. The B subunits of these toxins undergo acid-induced conformational changes which embed the B subunit in the endosomal membrane, forming a protein-conducting channel that subsequently allows A chain egress to the cytosol [1,2,3]. Other AB toxins have no pore-forming capacity and must therefore utilize an existing protein-conducting channel in the host endomembrane system for A chain passage to the cytosol. The endoplasmic reticulum (ER) is the only endomembrane organelle with such a channel, so these toxins must travel by vesicle carriers from the endosomes to the ER before A chain translocation to the cytosol can occur (Figure 1) [4,5,6,7]. This review will focus on the structural changes that accompany A chain movement from the ER to the cytosol. 

## 2. Order–Disorder–Order Transitions for AB-Type, ER-Translocating Toxins

Unfolding and refolding events are critical for productive intoxication with ER-translocating toxins. Ampapathi *et al.* [8] termed this process an order–disorder–order transition. The holotoxin-associated A chain moves from the cell surface to the ER in an ordered conformation. The unique environment of the ER lumen then promotes A chain dissociation from the rest of the toxin. The free A chain subsequently shifts to a disordered state which triggers its translocation to the cytosol via the quality control system of ER-associated degradation (ERAD). Extraction of the unfolded A chain through one or more membrane-spanning “translocon” pores requires the action of host proteins associated with the cytosolic face of the ER membrane. Most exported ERAD substrates are appended with polyubiquitin chains that serve as a molecular tag for degradation by the 26S proteasome, but the A chains of ER-translocating toxins lack the lysine residues targeted for ubiquitination and therefore persist in the cytosol long enough to modify their targets. The final disorder-to-order transition which places the translocated A chain in a folded, active conformation is facilitated by additional host–toxin interactions in the cytosol. Events related to each stage of the order–disorder–order transition will be considered below. Each subsection will begin with a general overview of the process, followed by specific information for each ER-translocating toxin. When no data are available for a particular toxin, it will not be considered in the corresponding subsection.

## 3. Holotoxin Disassembly

A/B subunit dissociation is a prerequisite for A chain translocation from the ER to the cytosol. For most ER-translocating toxins, the catalytic subunit is anchored to the rest of the toxin by a disulfide bond. Reduction of the disulfide bond can occur in the oxidizing environment of the ER and is likely facilitated by one or more ER-localized oxidoreductases [17,18,19,20,21,22]. Loss of the disulfide tether allows spontaneous or assisted separation of the reduced A chain from its holotoxin. The released A chain then shifts to an unfolded, translocation-competent conformation for export to the cytosol. Although disulfide bond reduction is a common event in the intoxication process, the exact mechanism differs for various ER-translocating toxins and is not even used by all of the toxins.

### 3.1. Cholera Toxin

**Figure 2:**
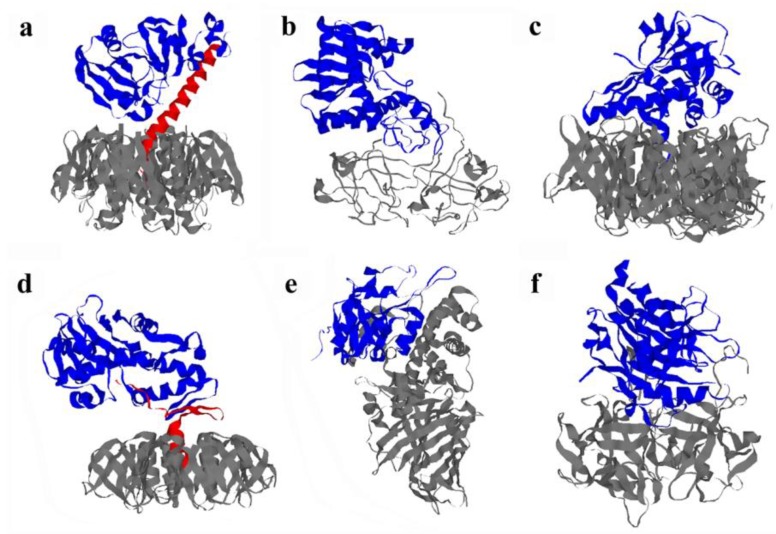
Structural organization of AB-type, ER-translocating toxins. (**a**) Ribbon diagram of cholera toxin (Ctx; PDB 1S5F, [33]). The A1 subunit is in blue; the A2 linker is red; and the B homopentamer is grey. The CtxB pentamer recognizes GM1 gangliosides on the host cell surface, while CtxA1 is an ADP-ribosyltransferase that elevates intracellular cAMP levels by activating the stimulatory α subunit of the heterotrimeric G protein; (**b**) Ribbon diagram of ricin toxin (Rtx; PDB 2AAI, [34]). RtxA is in blue, and RtxB is in grey. RtxB binds to a wide range of glycoproteins and glycolipids with terminal galactose residues, while RtxA is an *N*-glycosidase that inhibits protein synthesis by removing a specific adenine residue from the 28S rRNA; (**c**) Ribbon diagram of pertussis toxin (Ptx; PDB 1PRT, [35]). The catalytic S1 subunit is in blue, and the five subunits of the B pentamer (S2, S3, two copies of S4, and S5) are grey. PtxB can bind to a variety of glycoconjugates, while PtxS1 is an ADP-ribosyltransferase that elevates intracellular cAMP levels by locking the inhibitory α subunit of the heterotrimeric G protein in an inactive state; (**d**) Ribbon diagram of Shiga toxin (Stx; PDB 1DM0, [36]). The A1 subunit is in blue; the A2 linker is in red; and the B homopentamer is grey. The StxB pentamer binds to globoside Gb3 on the host cell surface, while StxA1 is an *N*-glycosidase that inhibits protein synthesis by removing a specific adenine residue from the 28S rRNA. The Stx family includes Stx from *Shigella dysenteriae* (pictured) and the Shiga-like toxins (Stx1, Stx2, and Stx2 isoforms) from *Escherichia coli*; (**e**) Ribbon diagram of *Pseudomonas aeruginosa* exotoxin A (EtxA; PDB 1IKQ, [37]). The catalytic moiety (domain III) is in blue, and the B moiety (domains I and II) is in grey. The B moiety of EtxA binds to the α-macroglobulin receptor/low density lipoprotein receptor-related protein on the host plasma membrane, while the A moiety of EtxA is an ADP-ribosyltransferase that inhibits protein synthesis through the modification of elongation factor 2; (**f**) Ribbon diagram of cytolethal distending toxin (Cdtx; PDB 1SR4, [38]). The catalytic CdtxB subunit is in blue, while the cell-binding CdtxA and CdtxC subunits are in grey. The cell-binding heterodimer binds to cholesterol and glycoconjugates, while the CdtxB subunit is a type I DNase that induces cell cycle arrest by causing double-stranded DNA breaks.

Cholera toxin (Ctx) is organized as an AB_5_ toxin with a catalytic A1 subunit, an A2 linker, and a ring-like B homopentamer [4] (Figure 2a). The A1 and A2 subunits are initially joined in a single CtxA polypeptide chain which is proteolytically nicked to generate an A1/A2 heterodimer that remains connected by a single disulfide bond between the *C*-terminus of the A1 subunit and the *N*-terminus of the A2 subunit. The A2 subunit extends into the central pore of the ring-like B pentamer and thus maintains extensive contacts with the B subunit. Numerous non-covalent interactions between CtxA1 and CtxA2 are also present. These non-covalent contacts are sufficient to preserve a stable, intact Ctx holotoxin even after reduction of the CtxA1/CtxA2 disulfide bond [23,24,25]. Disassembly of the reduced holotoxin results from an interaction with protein disulfide isomerase (PDI), an ER-localized oxidoreductase [26,27]. PDI-deficient cells are completely resistant to Ctx, which emphasizes the importance of PDI-mediated holotoxin disassembly to the intoxication process [26]. However, PDI is not required for reduction of the CtxA1/CtxA2 disulfide bond [21,28]. PDI can assist CtxA1/CtxA2 reduction [21,22], but its essential role for Ctx intoxication involves the physical displacement of reduced CtxA1 from CtxA2/CtxB_5_ [29]. This event was originally thought to involve the active unfolding of holotoxin-associated CtxA1 by PDI [27], but a recent biophysical analysis using isotope-edited Fourier transform infrared (FTIR) spectroscopy demonstrated that PDI does not unfold CtxA1 [26]. Instead, surprisingly, PDI itself unfolds upon contact with CtxA1. This phenomenon was documented by isotope-edited FTIR spectroscopy and far-UV circular dichroism (CD; [29]). The substrate-induced unfolding of PDI provides a structural explanation for the PDI-mediated separation of reduced CtxA1 from the Ctx holotoxin: the expanded hydrodynamic radius of unfolded PDI would act as a wedge to displace CTA1 from its non-covalent association with the rest of the toxin. In support of this model, PDI-mediated toxin disassembly does not occur when PDI unfolding is disrupted by treatment with the intramolecular cross-linker EDC or with ribostamycin, an inhibitor of PDI chaperone activity [29]. Ctx disassembly thus exploits a unique feature related to the chaperone function of PDI. 

### 3.2. Ricin Toxin

Ricin toxin (Rtx) is synthesized by the plant *Ricinus communis* as a single polypeptide chain that undergoes proteolytic nicking to generate a disulfide-linked RtxA/RtxB heterodimer [5] (Figure 2b). The single disulfide bond connecting RtxA to RtxB can be reduced by PDI [18,19,20]. Reduction is required for intoxication [19,20], but we have found a PDI-deficient cell line [30,31] exhibits wild-type sensitivity to Rtx [32]. In addition, PDI-immunodepleted cell extracts can still support reduction of the RtxA/RtxB disulfide bond [18]. These observations indicate PDI is not necessary for Rtx reduction or intoxication. Other oxidoreductases have been shown to reduce the RtxA/RtxB disulfide linkage, including the ER-localized thioredoxin-like transmembrane (TMX) protein [17,18]. Moreover, TMX-silenced cells are less susceptible to Rtx than their matched control cells [17]. These collective observations suggest that, unlike Ctx, reduction of the RtxA/RtxB disulfide bond is sufficient for Rtx disassembly. 

### 3.3. Pertussis Toxin

Pertussis toxin (Ptx) is an AB_5_ toxin that contains four different proteins in its ring-like B pentamer [6] (Figure 2c). The catalytic S1 subunit of Ptx contains an intramolecular disulfide bond but is anchored to its B heteropentamer via non-covalent contacts. The disruption of this non-covalent assembly is triggered by a structural change in the B pentamer resulting from ATP binding to the B subunit [39,40,41]. The ER is the only endomembrane organelle which contains ATP [42,43], so the Ptx holotoxin remains intact until it reaches the ER translocation site. Mutations which prevent ATP binding to the PtxB pentamer produce a holotoxin with no *in vivo* activity, which again emphasizes the importance of holotoxin disassembly to the intoxication process [6]. Reduction of the intramolecular PtxS1 disulfide bond is thought to occur after holotoxin disassembly [40] and results in activation of the latent PTS1 enzymatic activity [44]. 

### 3.4. Shiga Toxin

Shiga toxin (Stx) is an AB_5_ toxin that contains an enzymatic A1 subunit, an A2 linker, and a B homopentamer [7] (Figure 2d). Like Ctx, the A subunit of Stx is proteolytically nicked to generate a disulfide-linked A1/A2 heterodimer [45,46]. StxA1 is tethered to the rest of the toxin by this disulfide bond. Reduction of the StxA1/StxA2 disulfide bond by host oxidoreductases has not been examined, but proteolytic nicking of a Stx mutant lacking the StxA1/StxA2 disulfide bond results in dissociation of the StxA1 subunit from its holotoxin [47]. This indicates StxA1 will be released from the rest of the toxin upon reduction of the StxA1/StxA2 disulfide bond. 

### 3.5. Exotoxin A

*Pseudomonas aeruginosa* exotoxin A (EtxA) is a single chain AB toxin that is proteolytically nicked in acidified organelles of the host endomembrane system to generate a disulfide-linked heterodimer [48] (Figure 2e). Reduction of the disulfide bridge by PDI or other host oxidoreductases will liberate the *C*-terminal 37 kDa fragment, which is a prerequisite for its translocation to the cytosol [49,50]. However, the disulfide linkage in unnicked EtxA is not solvent-exposed [51]. The toxin must therefore undergo two conformational changes in order for host oxidoreductases to act upon the disulfide bond: (i) the toxin is nicked to its disulfide-linked heterodimeric state; and (ii) the EtxA heterodimer undergoes a structural shift that exposes the disulfide bond to solvent [49,52]. *In vitro*, this structural shift can be initiated by heating nicked EtxA to 50 °C. Incubation of nicked EtxA at 37 °C with proteins enriched in the membrane fraction of a cell extract will also cause a conformational shift [49]. This is an unusual process for toxin disassembly, as the A chains of ER-translocating toxins usually undergo substantial conformational changes only after separation from the holotoxin.

### 3.6. Summary

Covalent and non-covalent interactions maintain the stable architecture of an AB toxin until it reaches the ER translocation site. The unique physiology and protein content of the ER then facilitates holotoxin disassembly through the reduction of disulfide bonds, the destabilizing effect of ATP binding to the toxin, and/or the active displacement of the toxin A chain from its non-covalent association with the rest of the toxin. The released A chain then unfolds to a translocation-competent conformation that activates the ERAD system.

## 4. Intrinsic Instability of the Isolated Toxin A Chain

The organization of an AB holotoxin maintains the A subunit in a stable, folded conformation. This arrangement protects the holotoxin-associated A subunit from extracellular or endosomal/lysosomal proteases likely to be encountered in the host environment [47,53,54,55,56,57]. In addition to stabilizing the A chain, the B subunit is responsible for adhesion to the surface of a target cell and subsequent vesicle-mediated toxin delivery to the ER [4,5,6,7]. Holotoxin disassembly in the ER allows the free A chain to assume a disordered conformation which engages the ERAD system for export to the cytosol. As discussed below, unfolding of the dissociated A chain may occur spontaneously or may be assisted by an interaction with the ER membrane. Instability in the isolated A chain is a common property of ER-translocating toxins but is not seen in one toxin that utilizes an ERAD-independent translocation route.

### 4.1. Cholera Toxin

The AB_5_ configuration of the Ctx holotoxin is a stable complex that undergoes biphasic thermal transitions at 51 °C and 74 °C as assessed by differential scanning calorimetry (DSC). Denaturation of the CtxB pentamer is reflected by the 74 °C transition, which shifts to 95 °C when Ctx is bound to its GM1 ganglioside receptor [58]. Likewise, denaturation of the free CtxB pentamer occurs between 66–78 °C in the absence of GM1 and begins at 87 °C in the presence of GM1 [58,59,60,61]. The thermal denaturation of holotoxin-associated CtxA1, which is centered around 51 °C, is unaffected by holotoxin binding to GM1 [58]. However, the stability of the CtxA1/CtxA2 heterodimer is affected by its placement within the Ctx holotoxin: experiments using far-UV CD and FTIR spectroscopy found that a purified, disulfide-linked CtxA1/CtxA2 heterodimer exhibits a secondary structure transition temperature (*T*_m_; the midpoint of transition) of 43 °C and shifts to a disordered conformation between 40 and 46 °C [56,60]. A further loss of stability occurs upon reduction of the CtxA1/CtxA2 disulfide bond, as the free CtxA1 subunit has a disordered tertiary structure and partially perturbed secondary structure at the physiological temperature of 37 °C [56]. CtxA1 is also prone to aggregation at 37 °C [62]. Even the folded conformation of CtxA1 present at low temperature contains a substantial amount of poorly defined structure [8,60]. These collective observations demonstrate the labile CtxA1 polypeptide is stabilized to some extent by its disulfide linkage to CtxA2 and to a greater extent by its incorporation into the Ctx holotoxin. The ER-localized separation of CtxA1 from CtxA2/CtxB_5_ would thus result in the spontaneous unfolding of the free CtxA1 subunit. Unfolding of dissociated CtxA1 begins with a localized loss of structure in the *C*-terminal domain [63] and acts as a signal to engage the ERAD translocation machinery [63,64,65]. 

The inhibition of CtxA1 unfolding will prevent A chain translocation to the cytosol and, thus, productive intoxication. This phenomenon has been documented with three different experimental conditions: (i) treatment with glycerol; (ii) incubation at pH 6.5; and (iii) exposure to sodium phenylbutyrate (PBA). Glycerol is a chemical chaperone that forms a stabilizing hydration shell [66,67] around CtxA1 and other proteins. Exposure to acidic pH usually results in protein denaturation, yet CtxA1 is instead stabilized by mildly acidic pH (6.0–6.5) [63]. PBA is an ammonia scavenger that is used clinically to treat pediatric urea cycle disorders [68]. It also acts as a chemical chaperone that stabilizes CtxA1 through direct binding to the toxin [64]. As assessed by CD and fluorescence spectroscopy, all three experimental conditions blocked the thermal unfolding of CtxA1. These conditions also blocked the ER-to-cytosol translocation of CtxA1 and Ctx activity in cellular or intestinal models of intoxication [63,64,65]. In contrast, the conditions did not affect toxin transport from the cell surface to the ER, holotoxin disassembly, or toxin activity in the cytosol. Host mechanisms involved with Ctx intoxication (vesicle trafficking pathways, overall ERAD activity, and cAMP production) were likewise unaffected by the three conditions. These collective observations indicated that glycerol, acidic pH, and PBA each block Ctx intoxication by specifically inhibiting the thermal unfolding of free CtxA1 and its resulting ER-to-cytosol translocation. PBA and other drugs that act as protein stabilizers could thus represent a new class of anti-toxin therapeutic agents.

### 4.2. Ricin Toxin

The RtxA/RtxB heterodimer is a stable complex that retains full activity after a two hour incubation at 60 °C [69,70]. As determined by DSC, the melting temperature for holotoxin-associated RtxA at pH 7.5 is 74 °C [71]. In contrast, the isolated RtxA subunit has a melting temperature of 49 °C [71]. As assessed by CD and fluorescence spectroscopy, free RtxA shifts to an unfolded state between 40–50 °C and may resemble a molten globule between 42–45 °C [72,73,74,75,76]. RtxA therefore aggregates readily between 42–45 °C and is even prone to aggregation at 37 °C [77,78]. Additional studies using far-UV CD and fluorescence spectroscopy found that an interaction with the anionic phospholipids of the ER membrane results in a further, substantial loss of RtxA structure at physiological temperature. This interaction involves the hydrophobic *C*-terminal region of RtxA [79,80], which is only exposed after reduction and disassembly of the RtxA/RtxB heterodimer [18,19,81]. The interaction also requires a temperature-dependent conformational shift in the dissociated RtxA subunit, as the hydrophobic *C*-terminus of RtxA will insert into a membrane bilayer at 37 °C but not 30 °C [80]. Unfolding of RtxA to a translocation-competent conformation thus appears to involve a two-stage process: free RtxA first undergoes a minor conformational shift at 37 °C that promotes its intimate association with anionic phospholipid bilayers, which in turn produces a major conformational shift to a disordered state. This would subsequently identify membrane-associated RtxA as a substrate for ERAD-mediated translocation to the cytosol. After export, RtxA may remain associated with the cytoplasmic face of the ER membrane for the depurination of its ribosomal 28S rRNA target [82].

The importance of RtxA/phospholipid interaction for Rtx intoxication is highlighted by studies documenting a loss of *in vivo* (but not *in vitro*) activity for RtxA variants with either point mutations in the *C*-terminal domain or charged residues extending from the *C*-terminus [83,84,85,86,87]. In some cases, the loss of *in vivo* activity was directly linked to an inhibition of toxin translocation to the cytosol [85,87]. No studies have directly examined the predicted loss of toxin-phospholipid interactions for these RtxA variants, but the available data strongly suggest the unfolding resulting from ER membrane interaction with the *C*-terminus of RtxA is a pre-requisite for toxin delivery to the cytosol.

Other conditions that inhibit RtxA unfolding will also block its translocation to the cytosol and *in vivo* toxin activity. This was documented with a recombinant RtxA engineered to contain a stabilizing intramolecular disulfide bridge that was reduced with much lower efficiency than the RtxA/RtxB disulfide bond. The recombinant toxin exhibited wild-type activity *in vitro* but greatly attenuated activity against cultured cells [88]. Glycerol, which prevents the thermal unfolding of RtxA in either the absence or presence of anionic phospholipids [89], likewise blocks Rtx activity against cultured cells [90,91]. Acidic pH also prevents the temperature-induced loss of RtxA structure [72], the ER-to-cytosol export of RtxA [92], and Rtx intoxication [92,93]. PBA will stabilize RtxA in the absence but not the presence of anionic phospholipids [89]. As such, PBA does not protect cultured cells from ricin intoxication because the destabilizing effect of negatively charged phospholipids on RtxA structure is dominant over the stabilizing effect of PBA [89]. This again emphasized the importance of RtxA/phospholipid interactions for toxin translocation to the cytosol and indicated any structure-based therapeutics to block RtxA unfolding must overcome the additional destabilizing effect of anionic phospholipids. 

### 4.3. Pertussis Toxin

Both the Ptx holotoxin and the PtxB pentamer are stable complexes. The intact Ptx holotoxin undergoes denaturation in a single unfolding event centered around 63 °C as determined by DSC [94]. As visualized by atomic force microscopy, the PtxB subunit remains intact at temperatures up to 60 °C. Loss of the pentameric structure only occurs after heating PtxB to 70 °C for 10 min [95]. In contrast, the isolated PtxS1 subunit exhibits a tertiary structure *T*_m_ of 28.5 °C and a secondary structure *T*_m_ of 31 °C as established by near- and far-UV CD, respectively. Heating to 50 °C results in irreversible denaturation of the PtxS1 polypeptide [96]. Thus, as with CtxA1, PtxS1 would spontaneously shift to a disordered conformation upon its ER-localized displacement from the Ptx holotoxin. The unfolded PtxS1 subunit would then be treated as an ERAD substrate for export to the cytosol.

### 4.4. Shiga Toxin

Stx2 is not inactivated by pasteurization [97] and retains nearly full activity after a 1 h incubation at 60 °C [98]. The StxB pentamer is also heat-stable, exhibiting an unfolding transition around 88 °C as assessed by DSC [99]. No studies have yet examined the thermal stability of StxA1, although there are some parallels between StxA1 and RtxA: like RtxA, the hydrophobic *C*-terminus of StxA1 interacts with phospholipid bilayers [100,101,102] and is required for toxin activity against yeast or cultured cells but is dispensable for *in vitro* toxin activity [102,103]. In addition, the *C*-terminus of StxA1 is needed for toxin export from the ER to the cytosol [103]. Finally, both glycerol and acidic pH (but not PBA) protect cultured cells against Stx [89,104,105]. These collective observations suggest, albeit indirectly, that the free StxA1 polypeptide interacts with negatively charged phospholipids of the ER membrane to induce the unfolding event required for its ERAD-mediated translocation to the cytosol.

### 4.5. Exotoxin A

Both unicked and nicked EtxA holotoxins are stable entities, with secondary structure *T*_m_ values of 53 °C and 50 °C, respectively [49]. DSC experiments found that denaturation of EtxA occurs at 56 °C, whereas denaturation of the EtxA catalytic component (PE40) occurs at 45 °C [106]. Thus, as with other ERAD-exploiting toxins, EtxA disassembly represents a destabilizing event for the catalytic subunit. However, the relatively stable conformation of free PE40 suggests additional structural changes would be required to trigger its export to the cytosol. This could involve toxin-phospholipid interactions similar to those recorded for RtxA and StxA1, but additional supporting studies on the structure of PE40 are lacking. 

### 4.6. Cytolethal Distending Toxin

Cytolethal distending toxins (Cdtx) are produced by a range of Gram-negative pathogens and, with one exception, exhibit an AB_2_ structural organization [107,108] (Figure 2f). The catalytic subunit is designated CdtxB because of the location of the corresponding gene in the *cdt* operon. CdtxB generates double-stranded DNA breaks and is thus unique amongst the ER-translocating toxins in that it attacks a nuclear, rather than cytosolic, target. Only the CdtxB subunit exits the ER, but fluorescence spectroscopy and far-UV CD have shown CdtxB is a highly stable protein that maintains its native structure at temperatures up to 50 °C [91]. Furthermore, glycerol treatment does not confer cellular resistance to Cdtx [91]. These observations suggest an unfolding event is not required for CdtxB translocation. Interestingly, Cdtx is the only known ER-translocating toxin that does not utilize ERAD for export from the ER [109]. The thermally stable CdtxB subunit thus exits the ER by an ERAD-independent mechanism, whereas instability in the isolated toxin A chain appears to be a common property of ERAD-exploiting toxins. The stability, translocation mechanism, and intracellular target of CdtxB place it in a unique subcategory of ER-translocating toxins.

### 4.7. Summary

The unique environment of the ER promotes holotoxin disassembly at the site for A chain translocation to the cytosol. The holotoxin-associated A chain is held in a folded conformation, but it shifts to a disordered state upon its ER-localized separation from the rest of the toxin. Unfolding of the dissociated A chain occurs spontaneously for the less stable toxins and is assisted by an interaction with the ER membrane for the more stable toxins. In either case, the unfolded A chain is recognized as a substrate for ER-to-cytosol export by the ERAD system. An inhibition of A chain unfolding will block toxin access to the cytosol and productive intoxication, which suggests a new therapeutic strategy for the development of anti-toxin inhibitors.

## 5. ERAD Processing of the Toxin A Chain

Secretory proteins and resident proteins of the secretory pathway enter the endomembrane system from the ER. These proteins are usually delivered to the ER via co-translational insertion through the Sec61 pore. The proteins enter the ER in an unfolded state and subsequently attain a folded conformation with the assistance of ER-localized chaperones, glycosyltransferases, lectins, and oxidoreductases. Macromolecular assemblies are also formed with the assistance of these resident ER proteins. Aberrant folding and assembly will occur during protein maturation in the ER, but these misfolded/misassembled proteins can generate potentially toxic aggregates. Thus, if an aggregation-prone protein cannot be properly assembled and/or folded, it will be diverted to the ERAD pathway for export to the cytosol and degradation by the ubiquitin-proteasome system [110]. Interestingly, the same cohort of ER proteins involved with protein maturation is also involved with protein degradation [111].

When a misfolded/misassembled protein is identified by the ERAD system, it is exported to the cytosol through Sec61 and/or Hrd1 pores. The Sec61 translocon is a static, bidirectional pore involved with both protein import and protein export [112]. The Hrd1 translocon is a transient structure composed of Hrd1 and associated regulatory proteins such as Derlin-1 and SEL1L [113]. Various endogenous ERAD substrates have been shown to move from the ER to the cytosol through these two portals.

The dual functions of individual ERAD factors in protein folding and protein disposal has made a definitive assignment of ERAD function in toxin translocation problematic. However, as described below, the broad-spectrum toxin resistance seen in a panel of ERAD-defective cultured cells provides a strong functional link between ERAD activity and toxin translocation. In addition, numerous studies have identified roles for various ER chaperones and translocon components in ERAD-mediated toxin translocation. Although the general process is similar for all ERAD-exploiting toxins, the details vary for each specific toxin. 

### 5.1. Cholera Toxin

ERAD-defective cell lines with altered rates of ER-to-cytosol translocation are highly resistant to Ctx [114,115]. These cell lines were selected from a population of mutagenized CHO cells that were challenged with a combination of Rtx and EtxA, two ER-translocating toxins with different surface receptors, endocytic mechanisms, retrograde trafficking patterns, and cytosolic targets. We accordingly reasoned that the simultaneous acquisition of resistance to both toxins would most likely result from the disruption of a single event shared by both toxins—namely, ERAD-mediated export to the cytosol. In support of this prediction, the Rtx/EtxA-resistant cell lines exhibited unselected resistance Ctx but maintained wild-type sensitivity to the endosome-translocating diphtheria toxin (Dtx). One subset of toxin-resistant cell lines exhibited attenuated rates of ER-to-cytosol export for both CtxA1 and an endogenous ERAD substrate, the Z variant of α1-antitrypsin (α1AT-Z) [114]. Another subset of toxin-resistant cell lines exhibited accelerated rates of CtxA1 and α1AT-Z degradation [115]. Both types of ERAD defects were unselected phenotypes that limited the accumulation of cytosolic toxin and, thus, toxin activity in the cytosol. The genetic lesions responsible for these phenotypes have yet to be identified, but the available data demonstrated that proper ERAD activity is required for the Ctx intoxication process.

Individual ERAD factors have also been shown to play a role in CtxA1 translocation. PDI is responsible for displacing reduced CtxA1 from the Ctx holotoxin but does not appear to interact with free CtxA1, as the spontaneous unfolding of dissociated CtxA1 results in the release of its PDI binding partner [26]. The free, disordered CtxA1 polypeptide then appears to be treated as a typical ERAD substrate. Unfolded conformations of CtxA1 are recognized by ERdj3, an Hsp40 chaperone that masks the exposed hydrophobic amino acid residues of CtxA1. Expression of a dominant negative ERdj3 blocks the ER-to-cytosol export of CtxA1 and Ctx intoxication [116]. The RNAi-induced loss of function for ERdj5, another ER-localized Hsp40, also blocks CtxA1 translocation from the ER [117]. BiP, an ER-localized Hsp70 chaperone, prevents toxin aggregation at 37 °C and is required for CtxA1 export from ER-derived microsomes [62]. Substrates for the Hsp40 family of chaperones are often transferred to an Hsp70 chaperone [111,118,119], so it is likely that CtxA1 is passed from ERdj3 and/or ERdj5 to BiP as the toxin is processed by the ERAD system. ERp72 also plays a role in the ERAD processing of CtxA1: downregulation of this chaperone/oxidoreductase by RNAi increased the efficiency of CtxA1 delivery to the cytosol and produced a toxin-sensitive phenotype [28]. ERp72 thus appears to restrict CtxA1 access to the cytosol, possibly by inducing a gain-of-structure in the disordered toxin that would remove it from the ERAD translocation pathway. 

Both the Sec61 and Hrd1 translocons may be involved with CtxA1 export to the cytosol. An *in vitro* transcription/translation system for CtxA1 expression in ER-derived microsomes was used to demonstrate a physical interaction between CtxA1 and Sec61 [120]. However, a functional role for Sec61 in CtxA1 translocation has not been demonstrated to date. Derlin-1 can bind to CtxA1 and may play a supporting role in toxin translocation by delivering CtxA1 to the Hrd1 pore [121,122,123], but it has also been suggested that the inhibitory effects of dominant-negative Derlin-1 and Derlin-1 depletion on Ctx intoxication result from indirect, off-target effects [81,124]. This concern is a general caveat for experiments that alter endogenous levels of host proteins involved with the intoxication process. Overexpression of a truncated Hrd1, overexpression of an enzymatically inactive Hrd1, or depletion of Hrd1 by RNAi will also reduce the efficiency of CtxA1 delivery to the cytosol [122]. Thus, Sec61, Derlin-1, and Hrd1 could all play a role in CtxA1 translocation. Additional studies will be needed to assess the relative contributions of each protein to CtxA1 translocation, including possible redundant and/or cooperative functions.

### 5.2. Ricin Toxin

A functional role for ERAD in RtxA translocation was demonstrated with the ERAD-defective, Rtx-resistant CHO mutant cell lines [114,115]. Over-expression of an endogenous ERAD substrate has also been shown to decrease the amount of cytosolic RtxA, which suggested the efficiency of RtxA translocation was reduced by the diversion of ERAD activity to the over-expressed substrate [125]. The RNAi-induced down-regulation of ER degradation enhancing α-mannosidase I-like protein (EDEM) also reduced the cytosolic quantity of RtxA [125]. EDEM is an ER-localized lectin and ERAD component [126], but its interaction with Rtx does not require the N-linked carbohydrates of native RtxA or the galactose-specific binding pocket of RtxB [125]. The structural basis for carbohydrate-independent binding of EDEM to Rtx remains to be established, as does the interplay between Rtx and other ERAD factors within the mammalian ER. To date, functional roles in RtxA translocation have been established for BiP and GRP94, which is an ER-localized Hsp90 chaperone. The drug-induced inactivation of GRP94 protects cultured cells against Rtx challenge [77,127]. In contrast, BiP depletion by shRNA results in a toxin-sensitive phenotype [128]. This suggests RtxA may be removed from the ERAD export path due to its direct interaction with BiP, which is consistent with the low levels of cytosolic RtxA and the Rtx-resistant phenotype resulting from BiP overexpression in HEK293 cells [128]. The mammalian ERAD factors known to interact with RtxA thus treat the toxin as a typical misfolded substrate and attempt to either refold the toxin or export it to the cytosol for degradation. 

Both Sec61 and Hrd1 appear to function in the ER-to-cytosol export of RtxA. Rtx and RtxA can be co-immunoprecipitated with Sec61 from toxin-challenged mammalian cells [92,125]. Furthermore, strains of *Saccharomyces cerevisiae* with temperature-sensitive mutations in Sec61p fail to export ectopically expressed RtxA from the ER to the cytosol at the restrictive temperature for Sec61p-mediated dislocation [129]. A yeast ∆*hrd1* null strain also displays a reduced rate of RtxA export from the ER and withstands the cytotoxic effects of ER-localized RtxA better than the parental wild-type strain. Yeast null strains lacking the Hrd1p cofactors Hrd3p (SEL1L in mammals), Der1p (Derlin-1 in mammals), or Usa1p (Herp in mammals) exhibit phenotypes similar to the ∆*hrd1* strain [130]. In mammalian cells, the loss of SEL1L expression due to shRNA also reduces RtxA delivery to the cytosol and provides moderate protection against Rtx challenge (1.6–1.4 fold at the IC_50_ value) [82]. Thus, studies in both yeast and mammalian systems suggest a role for the Hrd1 complex in RtxA translocation. However, expression of a dominant negative Derlin-1 construct did not block RtxA delivery to the cytosol of (i) toxin-challenged mammalian cells [125] or (ii) transfected mammalian cells expressing an ER-localized RtxA construct [82]. Another study likewise reported that a ∆*der1* yeast strain does not display a defect in the ERAD processing of ectopically expressed, ER-localized RtxA [129]. Further studies will be required to clarify the role of Hrd1 co-factors in RtxA translocation and the relative contributions of Sec61 and Hrd1 complexes to the translocation event.

### 5.3. Shiga Toxin

CHO cells are naturally resistant to Stx [131], so the ERAD-defective CHO cell lines could not be used to assess the role of ERAD in StxA1 translocation. However, Vero cells overexpressing wild-type or truncated variants of ERdj3 are highly resistant to Stx [132,133]. Yu and Haslam further demonstrated, using an *in vitro* transcription/translation system with ER-derived microsomes, that the free StxA1 subunit co-immunoprecipitates with a complex of ERdj3, BiP, GRP94, and Sec61 [134]. Functional roles for BiP and GRP94 in StxA1 translocation have yet to be established, but these chaperones likely prevent the aggregation of disordered StxA1 and/or deliver StxA1 to the Sec61 channel for export to the cytosol.

Physical but not functional interactions between StxA1 and mammalian Sec61 have been demonstrated. In contrast, studies in yeast have documented a functional role for Hrd1 in StxA1 translocation: a StxA1 construct expressed directly in the ER was less toxic to a Δ*hrd1* null strain than the parental wild-type strain, and it entered the cytosol of the Δ*hrd1* null strain at an attenuated rate in comparison to the parental strain [135]. These observations were also recorded for a yeast strain expressing a catalytically inactive Hrd1p mutant and for three other yeast null strains, each lacking one component of the Hrd1 complex: Hrd3p, Der1p, or Usa1p. Yet no studies were performed with yeast Sec61p mutants. Likewise, no reported studies in mammalian cells have attempted to co-immunoprecipitate StxA1 with Hrd1. Given these considerations, it is possible that (i) StxA1 translocation does not involve the Sec61 channel; (ii) StxA1 can use both Sec61 and Hrd1 pathways to reach the cytosol; or (iii) StxA1 moves through the Sec61 pore in mammalian cells and the Hrd1 pore in yeast. 

### 5.4. Exotoxin A

Few studies have examined the role of ERAD in EtxA intoxication. ERAD-defective CHO cell lines are highly resistant to EtxA, which demonstrates proper ERAD function is required for *in vivo* EtxA activity [114,115]. However, interactions between individual ER chaperones and EtxA have not yet been established. An interaction between EtxA and the Sec61 translocon in ER-derived microsomes can be detected by co-immunoprecipitation and prevents the ER-to-cytosol export of immunogenic peptides [136]. Exogenously applied EtxA will also block calcium leakage through the Sec61 pore [137]. Collectively, these observations suggest the catalytic subunit of EtxA is recognized as an ERAD substrate for passage to the cytosol through the Sec61 translocon. 

### 5.5. Summary

Holotoxin disassembly will release an unstable, aggregation-prone A chain in the ER. The free A chain will accordingly be treated as a typical ERAD substrate. Binding to ER-localized chaperones will prevent A chain aggregation. Depending on which chaperone is involved, the toxin-chaperone interaction could result in either refolding of the A chain or delivery of the A chain to a translocon pore for export to the cytosol. All ERAD-exploiting toxins appear to engage in this process, although the details differ for each toxin. For example, GRP94 plays a functional role in the processing of RtxA but not CtxA1 [77,127]. These distinctions can be attributed to differences in the A chain structure, as several studies using A chain constructs expressed directly in the ER have demonstrated the B subunit is not required for toxin-ERAD interactions. Some reported differences between ERAD-exploiting toxins, or even differences reported for a single toxin, could also reflect species-specific toxin interactions. Given the unstable nature of the free A chain, the temperature of the experiment may also play an important role in data interpretation. For example, as discussed in the following sections, the free CtxA1 polypeptide is in an active, protease-resistant conformation at 25 °C but is in an inactive, protease-sensitive conformation at 37 °C [56,138]. Temperature-dependent effects on A chain structure could also influence the rate of toxin export from the ER and the efficiency of proteasomal degradation. Despite these caveats, it is clear that the ERAD system plays an active role in A chain translocation from the ER to the cytosol. Toxin-ERAD interactions within the ER will deliver the A chain to one or more translocon pores for export to the cytosol, but a host factor at the cytoplasmic face of the ER membrane will be required for A chain extraction from the ER. 

## 6. Toxin Extraction from the ER

ERAD substrates exit the ER in an unfolded state as they are threaded through a translocon pore to the cytosol. Efficient translocation thus requires an active mechanism to ensure unidirectional movement of the substrate to the cytosol. In most cases, the driving force for substrate export is provided by the hexameric AAA ATPase p97 (Cdc48p in yeast and plants) and its associated Ufd1/Npl4 heterodimeric complex. ERAD substrates passing through a translocon pore are thought to interact with the p97 complex at the cytosolic face of the ER membrane: p97 can bind directly to the substrate, while the Ufd1/Npl4 complex will bind to polyubiquitin chains added to the emerging protein by ER-associated ubiquitin ligases. ATP binding and hydrolysis is thought to enact a conformational change in p97 that provides the driving force for substrate translocation to the cytosol. Additional p97 co-factors interact with the proteasome, thereby facilitating the efficient degradation of exported substrates [139,140]. A few ERAD substrates are exported by a p97-independent mechanism, but the molecular details of this process are poorly characterized [141,142,143]. As discussed below, both p97-dependent and p97-independent pathways are utilized by various ER-translocating toxins.

### 6.1. Cholera Toxin

Experiments using RNAi or dominant negative constructs have found that p97 plays a minimal role in CtxA1 export to the cytosol [144,145,146]. In contrast, the depletion of Hsp90 by RNAi strongly inhibited Ctx intoxication and effectively blocked the ER-to-cytosol export of CtxA1. Identical results were obtained from cells treated with geldanamycin, an Hsp90 inhibitor. Geldanamycin also blocked Ctx-induced fluid accumulation in an ileal loop model of intoxication [127]. Hsp90 binds directly to the disordered conformation of CtxA1 and could possibly use its chaperone function to pull CtxA1 out of the ER: by coupling toxin translocation with refolding, Hsp90 would prevent the (re)folded CtxA1 protein from sliding back into the translocon pore. This process would provide the driving force for CtxA1 extraction from the ER. 

Hps90 also plays a role in the endosome-to-cytosol translocation of certain toxin A chains, and it has been suggested that translocation across the endosomal membrane depends on the refolding activity of Hsp90 [1]. To date, only ADP-ribosylating toxins (including Ctx) have been found to interact with Hsp90. It is therefore possible that Hsp90 interacts with a conserved motif found in all ADP-ribosylating toxins.

Interestingly, the depletion of Ufd1 or Npl4 by RNAi generates a toxin-sensitive phenotype [146]. This indicated the Ufd1/Npl4 complex acts, independently of p97, as a negative regulator of CtxA1 translocation. The role of Ufd1/Npl4 in CtxA1 translocation may also occur independently of the ubiquitination machinery, as (i) a recombinant CtxA1 construct without lysine residues (which serve as ubiquitin attachment sites) can still enter the cytosol to elicit a toxic response and (ii) inactivation of the ubiquitination machinery does not block the ER-to-cytosol export of CtxA1 [56,147]. Potential links between Hsp90 and Ufd1/Npl4, as well as the molecular mechanism of Ufd1/Npl4-regulated toxin translocation, await further study.

### 6.2. Ricin Toxin

Ectopic expression of RtxA in the ER of tobacco protoplasts was used to demonstrate a role for Cdc48p in toxin translocation. RtxA was trapped in the ER when co-expressed with a dominant negative Cdc48p, and this sequestration protected the transfected protoplasts from the cytotoxic action of RtxA [148]. Expression of a dominant negative p97 in transfected mammalian cells likewise conferred resistance to exogenously applied Rtx [123]. In contrast, the drug-induced inactivation of Hsp90 generated a Rtx-sensitive phenotype in cultured cells [77]. RtxA does not require ubiquitination for export to the cytosol, as intoxication is not inhibited by loss of the ubiquitination machinery or the absence of lysine residues in a recombinant RtxA subunit [76]. Additional studies demonstrated the lysine-less RtxA subunit can move from the ER to the cytosol of transfected tobacco protoplasts [148]. Thus, RtxA appears to utilize a p97/Cdc48p-dependent, but ubiquitin-independent, extraction mechanism.

In yeast, RtxA translocation may occur by an alternative mechanism involving the Rpt4 subunit of the 19S proteasome cap. Toxin export from the ER to the cytosol, as assessed by the turnover of an ectopically expressed RtxA, was not altered in yeast mutants lacking functional Cdc48p or Npl4p. Export also occurred independently of toxin ubiquitination: wild-type RtxA and a RtxA construct devoid of lysine residues exhibited the same rate of degradation. However, the turnover of ER-localized RtxA was attenuated in yeast lacking functional Rpt4p [130]. Inactivation of the Cim3p and Cim5p components of the 19S cap has also been shown to inhibit the turnover of ER-localized RtxA in yeast [129], but other proteins in the 19S cap are not required for either toxin export or degradation [130]. Rpt4p can partner with Cdc48p for the extraction of an endogenous substrate from the yeast ER [149], so it has been proposed that Rpt4p may serve as the extraction motor for RtxA translocation to the cytosol [81,130]. Other proteins in the 19S cap are also involved with the export of endogenous ERAD substrates [150], which is consistent with the data recorded for *cim3-1* and *cim5-1* mutants. This may represent another example of how the details of ERAD-mediated toxin translocation vary from one experimental system to another, as RtxA appears to use a p97/Cdc48p-dependent extraction system in plants and mammals but a 19S cap-dependent mechanism in yeast. 

### 6.3. Shiga Toxin

Few studies have examined the cytosolic extraction machinery involved with StxA1 translocation. In yeast, Cdc48p has been implicated in the ER-to-cytosol export of an ectopically expressed StxA1 subunit. Npl4p may couple StxA1 translocation to proteasomal degradation, but it appears that the functional pool of translocated StxA1 evades processing by Np14p. Furthermore, toxin ubiquitination is not required for export from the ER: a lysine-less variant of StxA1 could still exit the yeast ER to generate a cytotoxic effect [135]. It also appears that Hsp90 is not required for StxA1 translocation, as we have found the Hsp90 inhibitor geldanamycin does not affect Stx activity against cultured cells [151]. These collective observations suggest that StxA1 utilizes a p97/Cdc48p-dependent but ubiquitin-independent extraction mechanism to reach the cytosol. 

### 6.4. Summary

Unfolding of the dissociated A chain places it in a translocation-competent conformation that activates the ERAD system. Both p97-dependent and Hsp90-dependent pathways are exploited for A chain movement into the cytosol. Neither pathway requires ubiquitination of the toxin for substrate extraction. In fact, as discussed in the following section, the translocated A chain must actively avoid ubiquitination and the resulting proteasomal degradation in order to elicit a toxic response from the host cell. 

## 7. Toxin Evasion of Efficient Degradation by the Ubiquitin-Proteasome System

Most exported ERAD substrates are degraded by the cytosolic ubiquitin-proteasome system, although the rate of turnover varies widely from substrate to substrate. Accessible lysine residues in the ERAD substrate are appended with polyubiquitin chains during or after export to the cytosol, and this recruits the 26S proteasome to the tagged protein. Two macromolecular complexes form the 26S proteasome: a barrel-shaped proteolytic 20S particle, and a 19S cap which is positioned at one or both ends of the 20S barrel. The 19S cap binds to the polyubiquitinated substrate, unfolds the substrate in an ATP-dependent process, and threads the unfolded substrate into the central cavity of the 20S proteasome. Three separate catalytic activities positioned within the interior of the core 20S proteasome then degrade the target protein. The polyubiquitin chains are removed before proteolysis and recycled for further use. Since ubiquitin recognition and substrate unfolding functions are located in the 19S cap, the core 20S proteasome can only degraded a limited number of proteins in the absence of the 19S cap. These substrates are processed in a ubiquitin-independent manner and must already be in a disordered state in order to pass through the barrel of the 20S particle [152].

The original model of ERAD-mediated toxin translocation was formulated after it was noted the A chains of these toxins have an extreme arginine-over-lysine amino acid bias that is not found in the B chains of the same toxins [153]. Hazes and Read [154] further suggested in a landmark hypothesis paper that: (i) a hydrophobic patch at the *C*-terminus of the A chain allows the toxin to masquerade as misfolded protein for ERAD-mediated export to the cytosol; and (ii) the absence or paucity of lysine residues in the toxin A chain circumvents the ubiquitin-dependent degradation which usually accompanies ERAD-mediated translocation. This model has undergone some modifications over time but remains essentially intact. Structural studies have demonstrated the isolated A chain actually is an unfolded protein, although in some cases the hydrophobic *C*-terminal domain contributes to A chain unfolding via an interaction with the ER membrane. Additional studies confirmed the exported A chain avoids ubiquitin-dependent proteasomal degradation, but the unstable nature of the isolated A chain may leave it susceptible to ubiquitin-independent degradation by the core 20S particle. 

### 7.1. Cholera Toxin

CtxA1 has 15 arginine residues and two lysine residues, one of which is essential for optimal enzymatic activity [155]. A single lysine-for-arginine replacement at residue 172 in CtxA1 produced an attenuated toxin with just 30% of wild-type toxin activity. Co-incubation with a proteasome inhibitor restored wild-type levels of toxin activity to this R172K mutant, which indicated the loss of *in vivo* activity was due to its rapid clearance from the cytosol by the ubiquitin-proteasome system [147]. Other cell culture experiments have also demonstrated a direct correlation between toxin activity and the amount of cytosolic toxin: mutant CHO cells with accelerated rates of ERAD-mediated degradation are highly resistant to Ctx [115], and CtxA1 variants prone to proteasomal degradation are less potent than the wild-type toxin [156]. Pulse-chase experiments with transfected cultured cells have further documented the ubiquitin-independent proteasomal degradation of ER-localized CtxA1 constructs [56,157,158]. CtxA1-expressing cells accordingly generate higher levels of cAMP in the presence of a proteasome inhibitor than in the absence of an inhibitor [56]. However, the pathophysiological response to cAMP accumulation (*i.e*., chloride efflux) is not enhanced in the presence of a proteasome inhibitor [147]. Chloride efflux thus appears to reach a saturated response at sub-maximal levels of intracellular cAMP. As such, the elevated levels of cytosolic CtxA1 resulting from proteasomal inhibition contribute to higher levels of cAMP but not to additional chloride efflux. In contrast, efficient proteasome-mediated clearance of the cytosolic toxin can reduce both cAMP levels and chloride efflux [115,147,156]. Ctx must therefore avoid rapid proteasomal degradation in order to elicit cytopathic effects from the host cell.

*In vitro*, CtxA1 can be degraded in a ubiquitin-independent manner by the core 20S proteasome [56]. Degradation does not occur when CtxA1 is held in a folded conformation [65]. This suggests the *in vivo* ubiquitin-independent turnover of CtxA1 involves the 20S particle and results from the unstable nature of the free CtxA1 subunit. However, under most conditions, the extent of CtxA1 degradation by this mechanism is insufficient to block the cytopathic action of Ctx. 

### 7.2. Ricin Toxin

RtxA has 21 arginine residues and two lysine residues which do not appear to be targeted for ubiquitination in mammalian cells, as the lack of ubiquitination in a cultured cell line with a temperature-sensitive defect in ubiquitination did not alter the cytotoxic activity of Rtx [76]. Likewise, the absence of lysine residues in a recombinant RtxA subunit did not alter its *in vivo* activity against mammalian cells. In contrast, the addition of four lysine residues to another recombinant RtxA subunit produced a toxin with greatly attenuated *in vivo* activity against mammalian cells. Inactivation of the ubiquitin machinery or proteasome function restored the *in vivo* activity of this lysine-rich toxin [76], which indicated the evasion of proteasomal degradation is necessary for RtxA to manifest its cytotoxic activity. Consistent with this model, CHO mutants with accelerated rates of ERAD activity are more resistant to Rtx than their parental wild-type cells [115]. Furthermore, when treated with a proteasome inhibitor, toxin-challenged Vero cells contain more cytosolic RtxA and are more susceptible to intoxication than untreated controls cells [76,92]. Proteasome inhibitors also extend the half-life of a RtxA construct expressed directly in the ER of transfected mammalian cells [82]. These collective observations suggest RtxA is degraded in the cytosol by a ubiquitin-independent proteasomal mechanism. Under most conditions, however, the rate of turnover is not sufficient to counteract the activity of cytosolic RtxA. 

The ER-to-cytosol export and proteasomal degradation of RtxA has also been documented with ectopic expression of RtxA in the ER of tobacco protoplasts [148,159,160,161]. With this system, the efficiency of RtxA turnover was correlated to the number of lysine residues in the toxin: a recombinant RtxA subunit with no lysine residues was degraded with slower kinetics than wild-type RtxA, while a recombinant RtxA subunit with four additional lysine residues was degraded faster than wild-type RtxA. Furthermore, the rate of degradation corresponded to the relative *in vivo* activity of each construct: wild-type RtxA was more toxic than the lysine-rich RtxA variant but less toxic than the lysine-less RtxA variant [160]. Evasion of efficient degradation by the ubiquitin-proteasome system is therefore needed for optimal intoxication. These observations were generally consistent with data generated from mammalian systems, although the increased potency of the lysine-less RtxA in plant but not mammalian cells suggests ubiquitin-dependent proteasomal degradation may normally remove RtxA from plant but not mammalian cytosol. 

Two recent studies have challenged the role of the proteasome in RtxA degradation. It has been suggested that the sensitizing effect of proteasome inhibitors on Rtx intoxication is due to the previously unrecognized long-term toxicity resulting from an ~12 h exposure to the inhibitors themselves [162]. Given that elevated levels of cytosolic RtxA were detected after short-term (1–4 h) exposure to a proteasome inhibitor [82,92], it is possible that, soon after the initial toxin exposure, RtxA reaches a quantity of cytosolic toxin which represents a saturating amount for maximal toxicity. Inhibition of the proteasome could therefore increase the levels of cytosolic toxin without further sensitization to the toxin. Additional evidence for the proteasome-independent turnover of RtxA has been derived from studies in yeast: a yeast *pre1-1* mutant that is deficient in the chymotrypsin-like activity of the proteasomal core [163] does not display any defects in the turnover of ER-expressed RtxA and exhibits wild-type sensitivity to the toxin [130,162]. This does not, however, discount a role for the trypsin-like and caspase-like activities of the 20S proteasome in RtxA degradation. Finally, an RtxA subunit denatured with GdnHCl could not be degraded by the 20S proteasome *in vitro* at 37 °C [162]. Yet the structure of denatured RtxA is unlikely to mimic the structure of the disordered, and possibly ER-associated, cytosolic RtxA subunit that might be degraded by the 20S proteasome. The collective body of work on RtxA processing by the host cell has confirmed productive intoxication requires the evasion of ubiquitin-dependent proteasomal degradation. However, the mechanism by which RtxA is cleared from the host cell remains uncertain and could involve a currently unknown proteasome-independent mechanism. 

### 7.3. Pertussis Toxin

PtxS1 has 22 arginine residues and no lysine residues. Arginine-to-lysine substitutions at one, two, or three residues generated recombinant PtxS1 subunits with substantial *in vitro* activity but greatly attenuated *in vivo* activity. The loss of *in vivo* activity was apparently due to toxin degradation by the proteasome, as co-incubation of a proteasome inhibitor with these recombinant toxins restored the cytopathic effect of Ptx [164]. These observations indicated the evasion of ubiquitin-dependent proteasomal degradation by PtxS1 is essential for productive intoxication, yet the cytosolic pool of PtxS1 appears to be degraded by the proteasome nonetheless: we recently found with a surface plasmon resonance-based detection system [165] that the inhibition of proteasome function generates elevated levels of wild-type PtxS1 in the cytosol of toxin-challenged cells [166]. The *in vivo* turnover of cytosolic PtxS1 likely involves processing by the core 20S proteasome, which has been shown *in vitro* to degrade the disordered PtxS1 subunit at 37 °C. In contrast, the PtxB pentamer and the holotoxin-associated PtxS1 subunit were not susceptible to degradation by the 20S proteasome [96]. Thus, only the disordered conformation of free PtxS1 is a suitable substrate for the 20S proteasome. 

### 7.4. Shiga Toxin

StxA1 has 22 arginine residues and 2 lysine residues, but the role of this arginine-over-lysine amino acid bias in protecting StxA1 from ubiquitin-dependent proteasomal degradation has not yet been directly examined. Proteasomal degradation does appear to influence Stx activity, however: Vero cells treated with a proteasome inhibitor contained a greater pool of cytosolic StxA1 and were more sensitive to intoxication than the untreated control cells [167]. A StxA1 construct expressed in the yeast ER was likewise degraded in a proteasome-dependent manner [135]. These observations indicate StxA1 is treated as an ERAD substrate and degraded, albeit inefficiently, in the cytosol.

### 7.5. Cytolethal Distending Toxin

The catalytic CdtxB subunit from *Haemophilus ducreyi* does not utilize an ERAD-dependent translocation mechanism [109] but still exhibits the arginine-over-lysine amino acid bias present in the A chains of other ER-translocating toxins: CdtxB contains 24 arginine residues but only 2 lysine residues. Furthermore, the thermally stable CdtxB subunit is resistant to *in vitro* degradation by the 20S proteasome [91]. The translocated pool of CdtxB may therefore avoid proteasomal degradation altogether and consequently persist in the nuceloplasm/cytoplasm, although this possibility has not yet been examined. 

### 7.6. Summary

Most ERAD substrates are degraded in the cytosol by the ubiquitin-protesome system. The A chains of ER-translocating toxins avoid this fate because they lack the lysine residues required for ubiquitin conjugation. The evasion of efficient proteasomal degradation is essential for productive intoxication, as the extent of intoxication is linked to the amount of toxin in the cytosol. The unstable nature of the toxin A chain may render it susceptible to ubiquitin-independent degradation by the core 20S proteasome, but this mechanism cannot normally prevent the accumulation of cytosolic toxin and resulting manifestation of toxin activity. Refolding of the translocated A chain by host factors could also protect the toxin from processing by the 20S proteasome.

## 8. Refolding and Activation of the Cytosolic Toxin

The translocated A chain will enter the cytosol in an unfolded state and must evade proteasomal degradation long enough to elicit a cytopathic or cytotoxic response from the intoxicated cell. The translocated A chain must also regain a folded, active conformation to modify its cytosolic target. Refolding will not occur spontaneously due to the intrinsic instability of the isolated A chain. Host factors must therefore interact with the A chain in order to restore its structure and function. Each ER-translocating toxin appears to engage different host factors to accomplish A chain renaturation.

### 8.1. Cholera Toxin

As expected from its intrinsic instability, the free CtxA1 subunit displays little to no *in vitro* activity against its Gsα target or synthetic substrates at 37 °C [138,168]. ADP-ribosylation factor (ARF) proteins have long been known to serve as allosteric activators of CtxA1 [169], but they can only stimulate the activity of a folded CtxA1 subunit: the binding of ARF6 to disordered CtxA1 induces neither a gain-of-structure nor a gain-of-function for the toxin [170]. Instead, disordered CtxA1 interacts with Hsp90 and lipid rafts to attain a folded conformation with a basal level of activity at 37 °C that can be further stimulated by ARF. Hsp90 exhibits an extremely high affinity (7 nM *K*_D_) for CtxA1 [127] and does not appear to dissociate from CtxA1 after toxin extraction from the ER. Lipid rafts, where the G protein target of CtxA1 is located, display a “lipochaperone” property which promotes the refolding and activation of disordered CtxA1. This interaction requires the hydrophobic *C*-terminal domain of CtxA1 and is not seen with phospholipids mimicking the composition of the overall plasma membrane [168]. The sequential interaction of CtxA1 with Hsp90 and then lipid rafts appears to place the toxin in a conformation that facilitates its further activation by ARF proteins [171]. The refolding of translocated CtxA1 to an active conformation thus appears to be a complex process involving multiple factors in the host cytosol. 

### 8.2. Ricin Toxin

The RPT5 subunit of the 19S proteasome cap can prevent the aggregation of denatured RtxA [162]. Hsc70 and Hsp90 chaperones also prevent the aggregation of cytosolic RtxA. However, further processing of cytosolic RtxA depends upon which co-chaperones are recruited to the Hsc70- or Hsp90-bound toxin: the turnover of RtxA is linked to its interaction with Hsp90 and Hsp90 co-chaperones, whereas the activation of translocated RtxA involves Hsc70 and a separate set of co-chaperones. The drug-induced loss of Hsp90 function consequently sensitizes cells to Rtx, while the drug-induced inhibition of Hsc70 leads to toxin resistance [77]. It remains to be determined whether Hsc70 promotes the cytosolic activity of RtxA through direct renaturation or indirect protection from degradation. RtxA may not require chaperone-mediated refolding, as an interaction with its ribosome target has been shown to induce a gain-of-function in the disordered conformation of RtxA [72]. 

### 8.3. Pertussis Toxin

Unlike CtxA1, the free PtxS1 subunit exhibits *in vitro* enzymatic activity at 37 °C in the absence of host proteins or lipids [172]. This difference can be attributed to the structural effect of NAD on PtxS1: NAD, the donor molecule for the PtxS1-catalyzed ADP-ribosylation of Giα, prevents PtxS1 from shifting to a protease-sensitive conformation at physiological temperature. The stabilizing effect of NAD was not observed for CtxA1, which also uses NAD as the donor molecule for its ADP-ribosylation of Gsα [96]. NAD is present in the cytosol but not the ER lumen [173], so it would not interact with PtxS1 until after the initial ER-localized unfolding event had triggered toxin export from the ER to the cytosol. Future structural studies should determine whether host factors such as Hsp90 and lipid rafts also contribute to the refolding of cytosolic PtxS1.

### 8.4. Summary

The unstable nature of the isolated A chain facilitates its ERAD-mediated export to the cytosol, but toxin instability also presents an obstacle to productive intoxication: the translocated A chain will not spontaneously return to a folded state, so it must rely upon host factors to regain an active conformation. These host factors include chaperones, donor molecules for the enzymatic reaction catalyzed by the toxin, and the target of the toxin itself. For the latter two examples, the toxin A chain bears some similarity to the class of intrinsically disordered proteins which only gain a functional conformation in the presence of their physiological binding partner(s) [174].

## 9. Conclusions

The intact AB holotoxin is delivered from the cell surface to the ER in a series of vesicle-mediated transport steps collectively termed retrograde transport. Holotoxin disassembly, which is facilitated by the unique physiology and protein content of the ER, releases the structural constraints on A chain unfolding. The A chain thus maintains an ordered, stable conformation until it reaches the ER translocation site. Spontaneous or assisted unfolding of the free A chain then triggers the ERAD system. Although toxin instability activates the ERAD translocation mechanism, it also presents co- and post-translocation obstacles for productive intoxication: the disordered A chain must be actively extracted to the cytosol through a translocon pore and must interact with host factors to regain an ordered, active conformation in the cytosol. The translocated A chain must also avoid proteasomal degradation (both ubiqutin-dependent and ubiquitin-independent) long enough to elicit a toxic effect through the modification of its cytosolic target. All ERAD-exploiting toxins follow this general order–disorder–order process, but the details vary for each toxin. Ongoing studies should continue to elucidate the role of A chain instability in toxin translocation, thereby providing new insights into both toxin pathogenesis and host cell biology.
